# Clinical efficacy and safety of acupuncture in modulating autonomic nervous function: a meta-analysis of randomized controlled trials

**DOI:** 10.3389/fnins.2025.1694110

**Published:** 2025-10-28

**Authors:** Ying-zhi Ma, Pu-yao Zhang, Tao Du, Zi-chen Wang, Tian-duo Wang, Ai-hui Fu, Zhao-mei-zi Wang, Tie-ming Ma, Wei Zhang

**Affiliations:** ^1^Liaoning University of Traditional Chinese Medicine, Shenyang, Liaoning, China; ^2^The First Affiliated Hospital of Liaoning University of Traditional Chinese Medicine, Shenyang, Liaoning, China

**Keywords:** acupuncture, autonomic nervous system, heart rate variability, meta-analysis, randomized controlled trial, safety

## Abstract

**Background:**

The autonomic nervous system (ANS) plays a pivotal role in maintaining physiological homeostasis, and its dysfunction is implicated in various chronic disorders. Current pharmacological and neuromodulatory interventions are constrained by limitations such as adverse effects and invasiveness. Acupuncture, a cornerstone of complementary and alternative medicine (CAM), demonstrates potential for bidirectionally modulating ANS function, yet systematic evidence remains scarce.

**Methods:**

A meta-analysis was conducted on randomized controlled trials (RCTs) retrieved from PubMed, Embase, CNKI, Wanfang, and CENTRAL databases from their inception until 1 August 2025. Data on baseline characteristics, heart rate variability (HRV) parameters (standard deviation of normal-to-normal intervals (SDNN), low frequency (LF), high frequency (HF), LF/HF), and adverse events were extracted. Analyses were performed using random-effects models.

**Results:**

Ten RCTs comprising 744 patients were included. Acupuncture significantly improved SDNN. True effect sizes for LF and HF suggested potential differences, but considerable uncertainty was evident. The incidence of adverse events was low, with no serious events reported.

**Conclusion:**

Acupuncture may confer modest improvements in ANS function, particularly evidenced by SDNN enhancement, and exhibits a favorable safety profile. However, the evidence is constrained by heterogeneity and methodological limitations, necessitating further validation through high-quality studies.

## Background

The autonomic nervous system (ANS) constitutes a fundamental regulatory mechanism for physiological homeostasis, comprising two primary branches: the sympathetic nervous system (SNS), which mediates “fight-or-flight” responses, and the parasympathetic nervous system (PNS), responsible for “rest-and-digest” functions (Wang et al., 2025). The ANS exerts multilevel control over cardiovascular, metabolic, and immune functions via pathways such as the neuro-cardiac axis. ANS imbalance—characterized by sympathetic overactivation or diminished parasympathetic tone—is intimately associated with a spectrum of pathologies, including chronic kidney disease (Zoccali et al., 2025), heart failure (Ziegler et al., 2025), and type 2 diabetes mellitus (Zsombok et al., 2024). For instance, ANS dysregulation in hepatic glucose metabolism can precipitate excessive gluconeogenesis, thereby exacerbating diabetic progression. Furthermore, the ANS modulates systemic inflammatory responses through vagal and adreno-medullary dopaminergic pathways, playing a critical role in pathological processes such as sepsis (Abe et al., 2023; Yang et al., 2023).

Current primary modalities for ANS modulation encompass pharmacological interventions and neuromodulation techniques, both of which present significant limitations. While pharmacological agents (e.g., beta-blockers) can mitigate sympathetic hyperactivation, they may induce adverse effects such as bradycardia and lack precision in targeting specific neural pathways (Lambiase et al., 2023; Costa et al., 2024). Neuromodulation techniques (e.g., vagus nerve stimulation) demonstrate promise in managing inflammatory conditions; however, their applicability is restricted by invasiveness and high costs (Wachsmuth et al., 2025). Moreover, perioperative ANS dysregulation frequently contributes to cardiovascular instability and heightened infection risk, yet existing methods fall short of facilitating dynamic modulation (Pan et al., 2025). These approaches are generally hampered by insufficient target specificity, notable side effects, or operational complexity. Consequently, the identification of novel therapeutic strategies for ANS dysregulation is of paramount importance.

According to the holistic theory of Traditional Chinese Medicine, the preservation of human health hinges upon the equilibrium of Yin and Yang and the harmony among internal organs. Meridians serve as crucial conduits interconnecting the internal and external bodily environments, thereby underpinning homeostasis. The principal objective of acupuncture therapy is to “dredge the meridians and regulate Qi and blood,” which manifests as the alleviation of discomfort (e.g., pain) and the overall amelioration of patient status. Clinical investigations indicate that chronic conditions such as osteoarthritis are frequently concomitant with ANS dysregulation (e.g., sympathetic hyperreactivity). Acupuncture may specifically address ANS imbalance by attenuating sympathetic excitability to reduce pain sensitivity while concurrently enhancing parasympathetic tone to promote tissue repair (Yeater et al., 2022). Furthermore, varying stimulation parameters can selectively modulate distinct ANS pathways. For example, low-frequency electroacupuncture at ST36 activates vagus nerve-mediated anti-inflammatory pathways, whereas high-frequency stimulation may modulate local immune microenvironments via sympathetic mechanisms (Liu et al., 2021). This bidirectional regulatory capacity of acupuncture appears conceptually aligned with the homeostatic functions of the ANS, potentially offering a low-risk, high-benefit therapeutic strategy for ANS imbalances arising from diverse pathologies. Nevertheless, a systematic, comparative, evidence-based evaluation of acupuncture against alternative interventions for ANS modulation is presently lacking.

Thus, this meta-analysis aims to compare the effects and safety of acupuncture vs. other therapeutic interventions on autonomic nervous system function in patients based on data synthesized from randomized controlled trials (RCTs).

## Methods

### Search strategy

This review followed the PRISMA guidelines. We searched PubMed, Embase, CNKI, Wanfang, and the Cochrane Central Register of Controlled Trials (CENTRAL) databases from inception until 1 August 2025. Reference lists of retrieved articles were also screened for additional studies. The study protocol was registered on PROSPERO (CRD420251121986).

### Selection criteria and data extraction

#### Inclusion criteria

Randomized controlled trials (RCTs) focusing on heart rate variability (HRV) as an outcome were considered, without restrictions on the health status or specific diseases of participants. Studies investigating any acupuncture technique and comparing it with other interventions were eligible. Articles in all languages were included.

#### Exclusion criteria

The exclusion criteria encompassed studies involving animal experiments or laboratory research; outcomes not explicitly reported; absence of data for at least one of the key HRV parameters (SDNN, LF, HF, and LF/HF); and use of interventions other than acupuncture in the experimental group.

Two reviewers independently screened the literature, extracted data, and assessed the risk of bias (RoB) using a predefined form. Disagreements were resolved through discussion or third-party consultation. Data collected included author, year, country, trial ID, patient demographics, intervention details, and outcomes. The RoB was evaluated using the Cochrane RoB 2.0 tool (Higgins et al., 2019). For studies with multiple groups, data from the non-intervention groups were excluded.

### Statistical analysis

The study, patient, and intervention characteristics were summarized. For dichotomous outcomes, risk ratios (RRs) with 95% confidence intervals (CIs) were calculated, whereas for continuous outcomes, mean differences (MDs) with 95% CIs were reported. Random-effects models were applied due to expected heterogeneity. Continuous outcome scales were standardized. Evidence certainty was rated using the GRADE framework. Sensitivity analyses were conducted by sequentially excluding individual studies. Publication bias was assessed using funnel plots.

## Results

### Study selection

**Figure 1:**
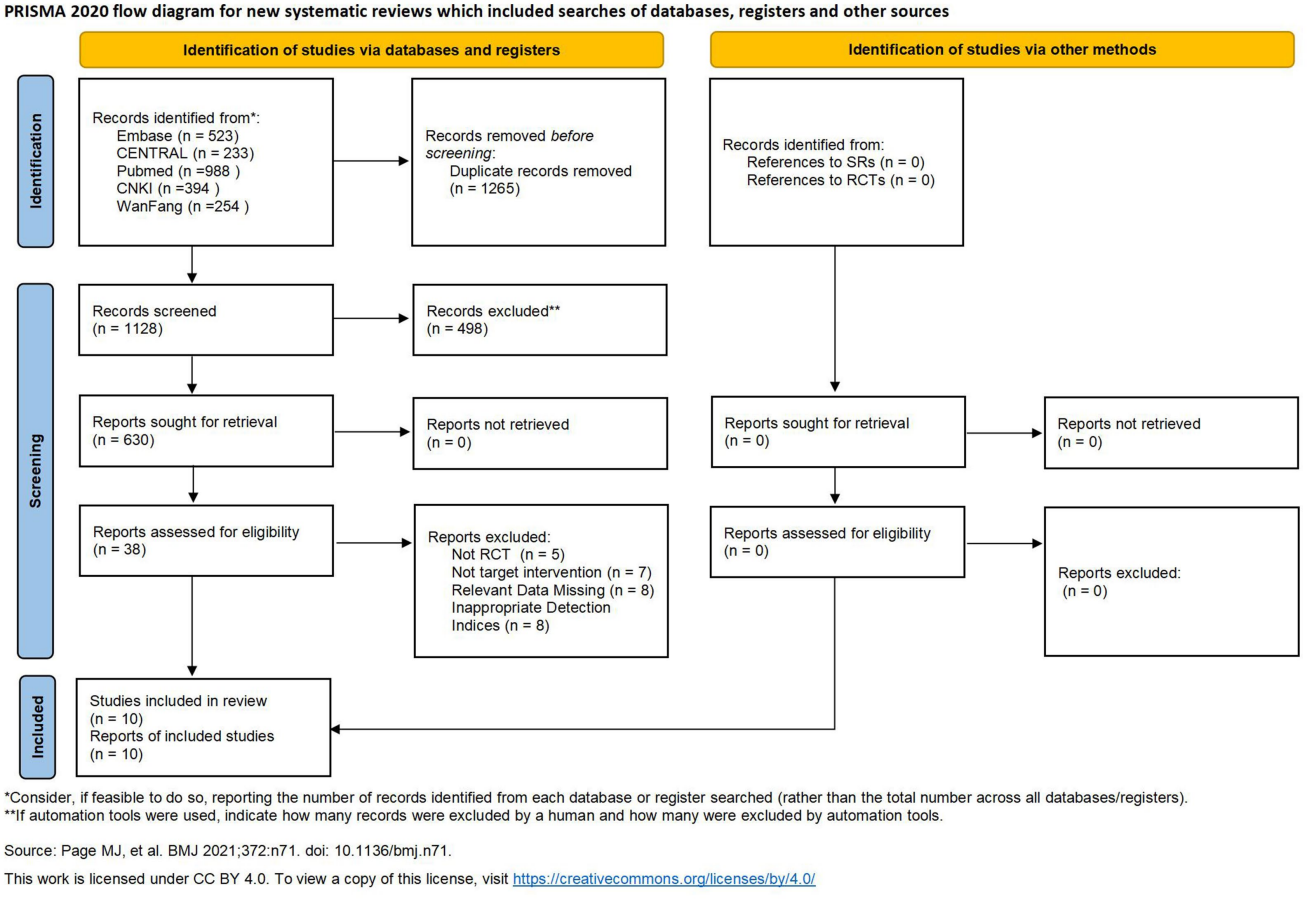
PRISMA flowchart of literature selection.

The PRISMA flow diagram detailing the study selection process is presented in Figure 1 (Page et al., 2021). Initially, 2,393 records were identified (PubMed = 988, Embase = 523, CNKI = 394, Wanfang = 254, and CENTRAL = 233). After removing duplicates and screening titles and abstracts, 38 studies underwent full-text assessment. Ultimately, 10 articles met the inclusion criteria and were incorporated into the meta-analysis (Guo et al., 2019; Di et al., 2023; Xinyu et al., 2024; Qu et al., 2015; Wang and Xiao, 2024; Niu et al., 2015; Zhang et al., 2017; Liao et al., 2024; Huang et al., 2020; Singh et al., 2024).

### Study characteristics

Among the included RCTs, nine were conducted in China and one in India; all evaluated the therapeutic effects of acupuncture on autonomic function. All 10 studies were published (six in Chinese and four in English). None were registered on ClinicalTrials.gov.

A total of 744 patients were enrolled (374 in the acupuncture group and 370 in the control group). The characteristics of the included studies are summarized in Table 1.

**Table 1:** Characteristics of the included studies.

Study	N (intervention/control)	Disease	Age, mean (SD), years	Sex (male/female)	Disease duration, mean (SD), months	Treatment duration
Guo et al. (2019)	60/60	Primary insomnia	48.67 ± 3.14	26/94	5.30 ± 1.60	4 weeks (once every other day, 12 times)
Di et al. (2023)	16/16	Post-percutaneous coronary intervention	68.3 ± 13.1	14/18	0.36 ± 0.02	2 weeks (14 days + 2 rest days between 2 cycles)
Xinyu et al. (2024)	51/53	Hyperarousal state in chronic insomnia	53.93 ± 12.39	21/83	79.2 ± 82.4	4 weeks (once every other day, 14 times)
Qu et al. (2015)	29/24	Chronic heart failure	64.55 ± 4.39	23/30	Information was missing	4 weeks (28 days +2 rest days between 2 cycles)
Wang and Xiao (2024)	30/30	Perimenopausal syndrome	50.5 ± 2.0	Sex information was missing	32.0 ± 10.7	30 min
Niu et al. (2015)	60/60	Post-stroke depression	59.44 ± 9.70	65/55	7.05 ± 1.52	6 weeks
Zhang et al. (2017)	40/40	Post-stroke depression	58.2 ± 6.0	37/43	7.95 ± 3.3	8 weeks (once every other day, 24 times)
Liao et al. (2024)	37/36	Post-hemodialysis	59.97 ± 12.38	26/47	93.96(40.56–159)	12 weeks (once every other day, 36 times)
Huang et al. (2020)	31/31	Elderly hypertension	71.87 ± 5.65	21/41	95.4 ± 39.36	12 weeks (once every other day, 36 times)
Singh et al. (2024)	20/20	Hypertension	54.00 ± 7.76	18/22	80.88 ± 75.72	10 days

### Estimation of quality

The methodological quality of the included randomized controlled trials was assessed using the Cochrane Risk of Bias tool (RoB 2.0), which systematically evaluates the risk of bias across five core domains:

Randomization process: All studies stated that random assignment was used. Random sequence generation methods included computer-generated random sequences and random number tables. However, the majority of studies did not provide sufficient details regarding the method of allocation concealment, making it impossible to exclude the possibility that investigators or participants could foresee the allocation sequence before enrollment. These were therefore judged as having “some concerns” (Guo et al., 2019; Qu et al., 2015; Wang and Xiao, 2024; Niu et al., 2015; Huang et al., 2020; Singh et al., 2024). Four studies explicitly used appropriate allocation concealment measures, such as central randomization (SMS) or sealed opaque envelopes, effectively preventing selection bias, and were rated as “low risk” (Di et al., 2023; Xinyu et al., 2024; Zhang et al., 2017; Liao et al., 2024).

Deviations from intended interventions: Due to the specific nature of acupuncture procedures, none of the studies could blind practitioners or participants. This inherent limitation may introduce performance bias, leading to a “high risk” rating for all studies in this domain.

Missing outcome data: All included studies reported complete or nearly complete outcome data, with low attrition rates and appropriately handled missing data, posing no significant threat to the validity of the results. This domain was therefore rated as “low risk” across all studies.

Measurement of the outcome: The majority of studies did not explicitly report whether outcome assessors were blinded to group allocation, creating a potential risk of detection bias and resulting in a “high risk” rating (Guo et al., 2019; Qu et al., 2015; Wang and Xiao, 2024; Niu et al., 2015; Huang et al., 2020; Singh et al., 2024). Four studies explicitly stated that outcome assessors were blinded to group assignment and were rated as “low risk” (Di et al., 2023; Xinyu et al., 2024; Zhang et al., 2017; Liao et al., 2024).

Selection of the reported result: None of the studies provided a pre-specified analysis plan in their publications or trial registration records, making it impossible to fully rule out selective outcome reporting. This domain was therefore judged as having “some concerns” across all studies.

**Figure 2:**
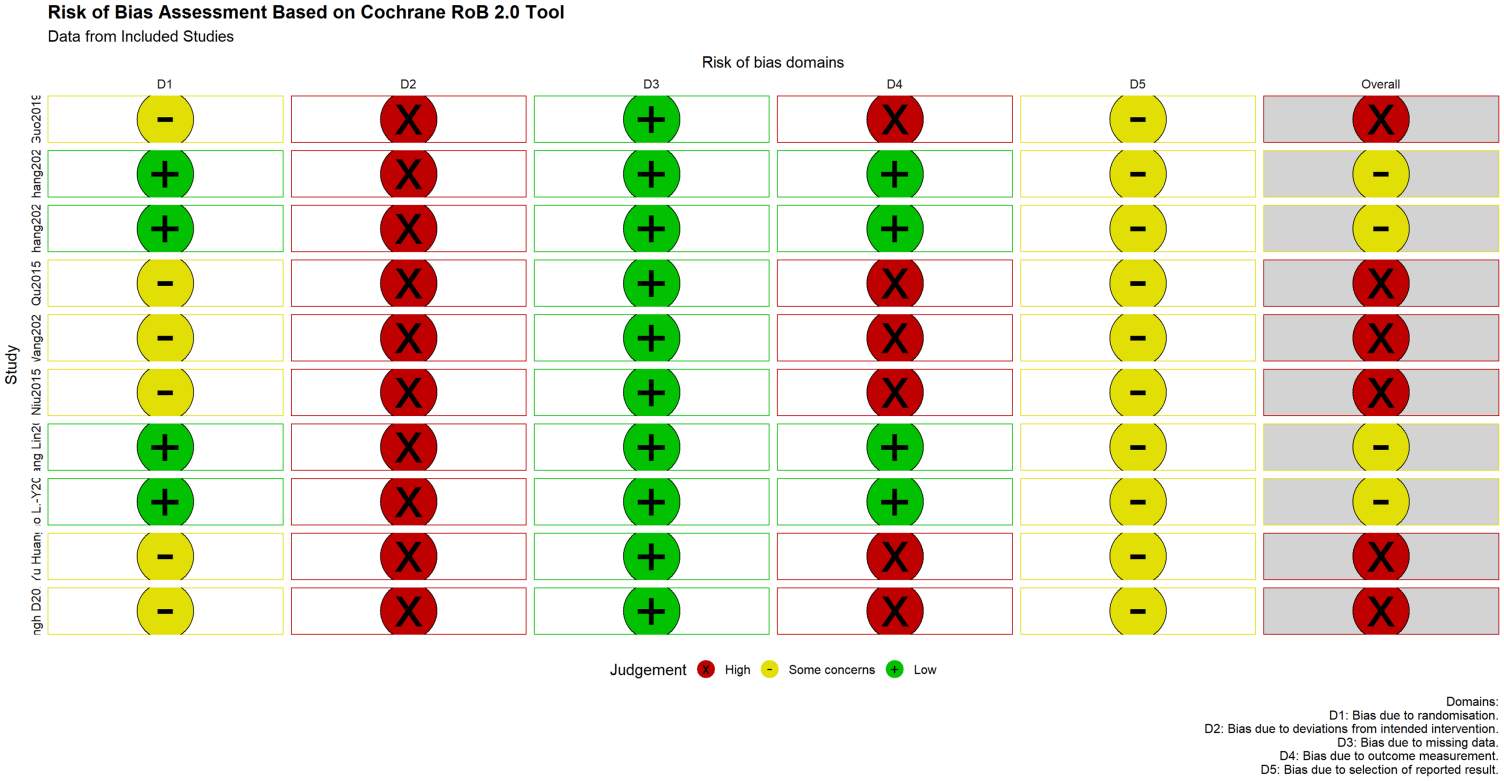
Risk of bias assessment for each study based on the Cochrane RoB 2.0 tool.

The overall assessment indicated that six of the ten included studies were at “high risk” of bias, while four raised “some concerns” (Figure 2).

### Treatment efficacy

**Figure 3:**
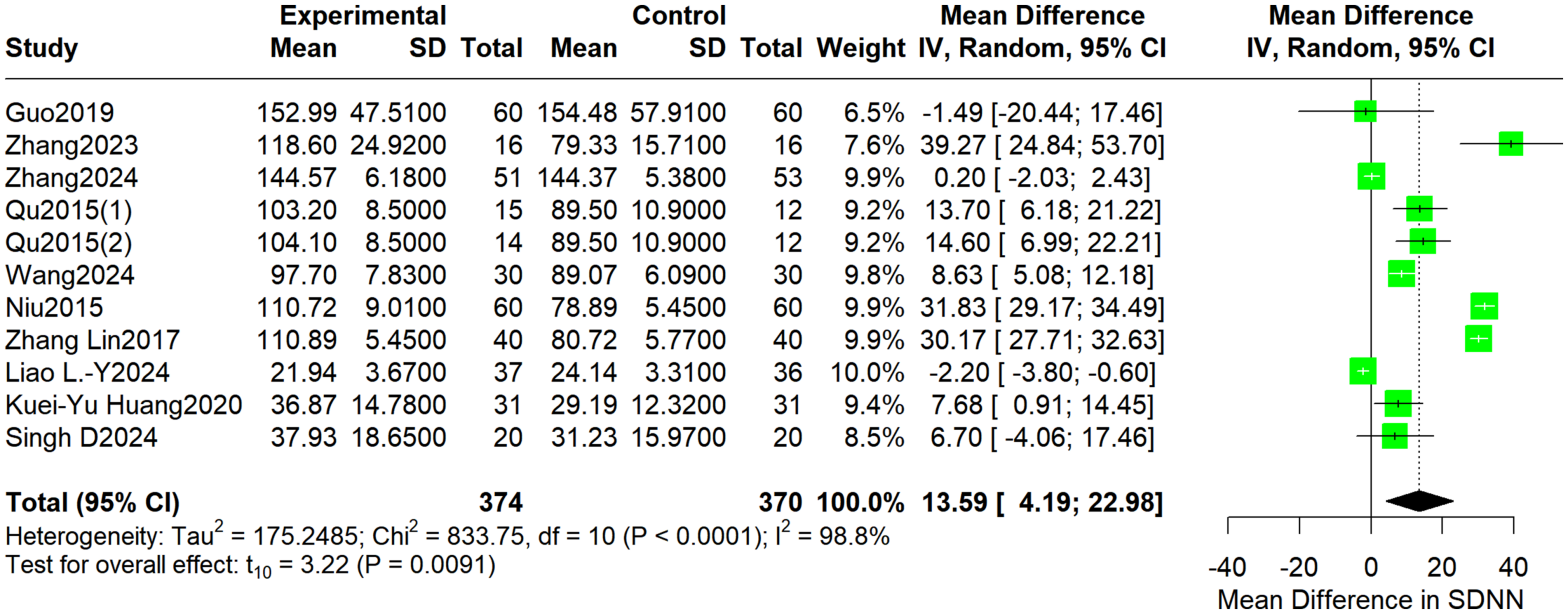
Forest plot depicting differences in SDNN between acupuncture treatment and control interventions.

Regarding HRV outcomes, nine studies reported short-to-medium-term results (2–6 weeks), and one study reported immediate effects (single session). For SDNN, the pooled MD was 13.59 (95% CI: 4.19 to 22.98; I^2^ = 98.8%, *p* = 0.0091) (Figure 3).

**Figure 4:**
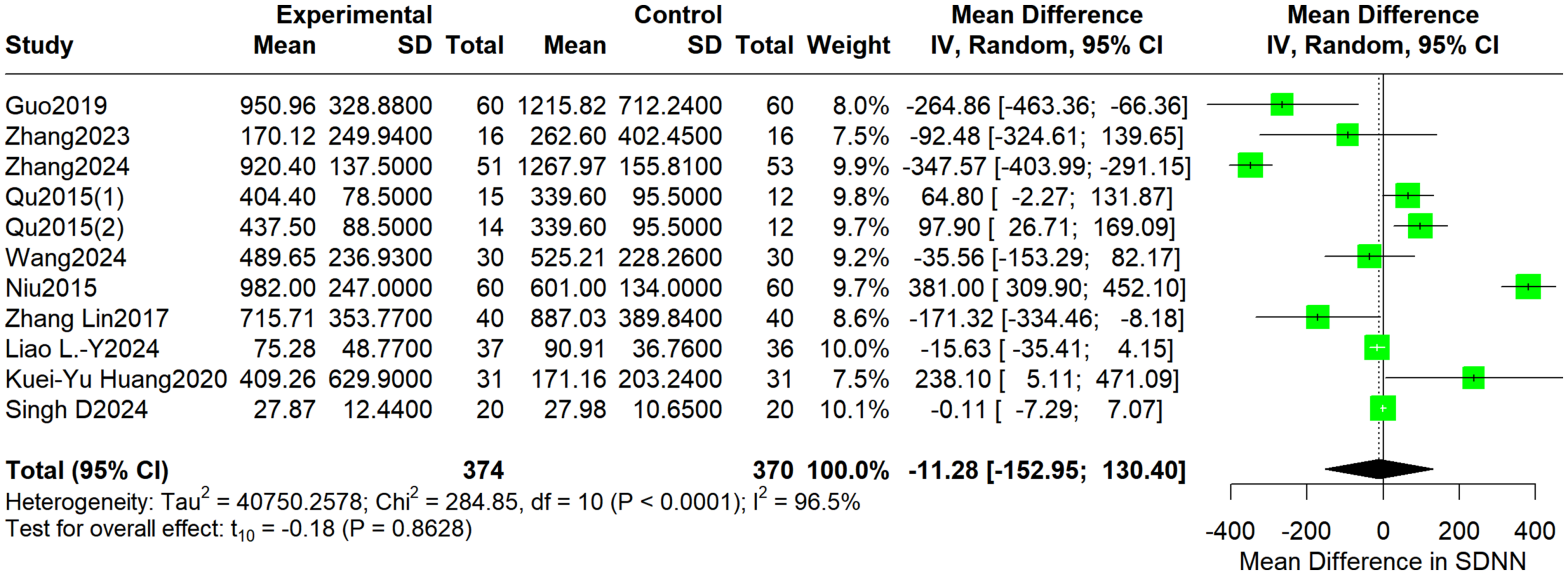
Forest plot depicting differences in LF between acupuncture treatment and control interventions.

For LF, the pooled MD was −11.28 (95% CI: −152.95 to 130.40; I^2^ = 96.5%, *p* = 0.8628) (Figure 4).

**Figure 5:**
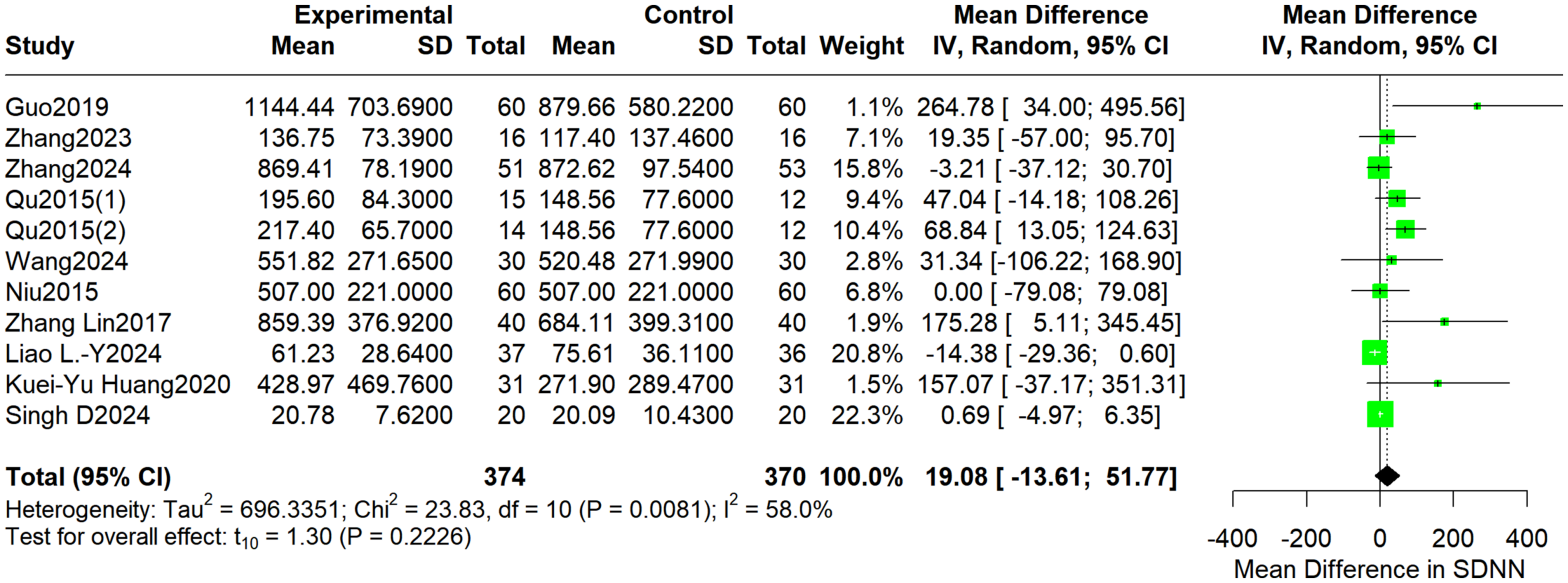
Forest plot depicts differences in HF between acupuncture treatment and control interventions.

For HF, the pooled MD was 19.08 (95% CI: −13.61 to 51.77; I^2^ = 58.0%, *p* = 0.2226) (Figure 5).

**Figure 6:**
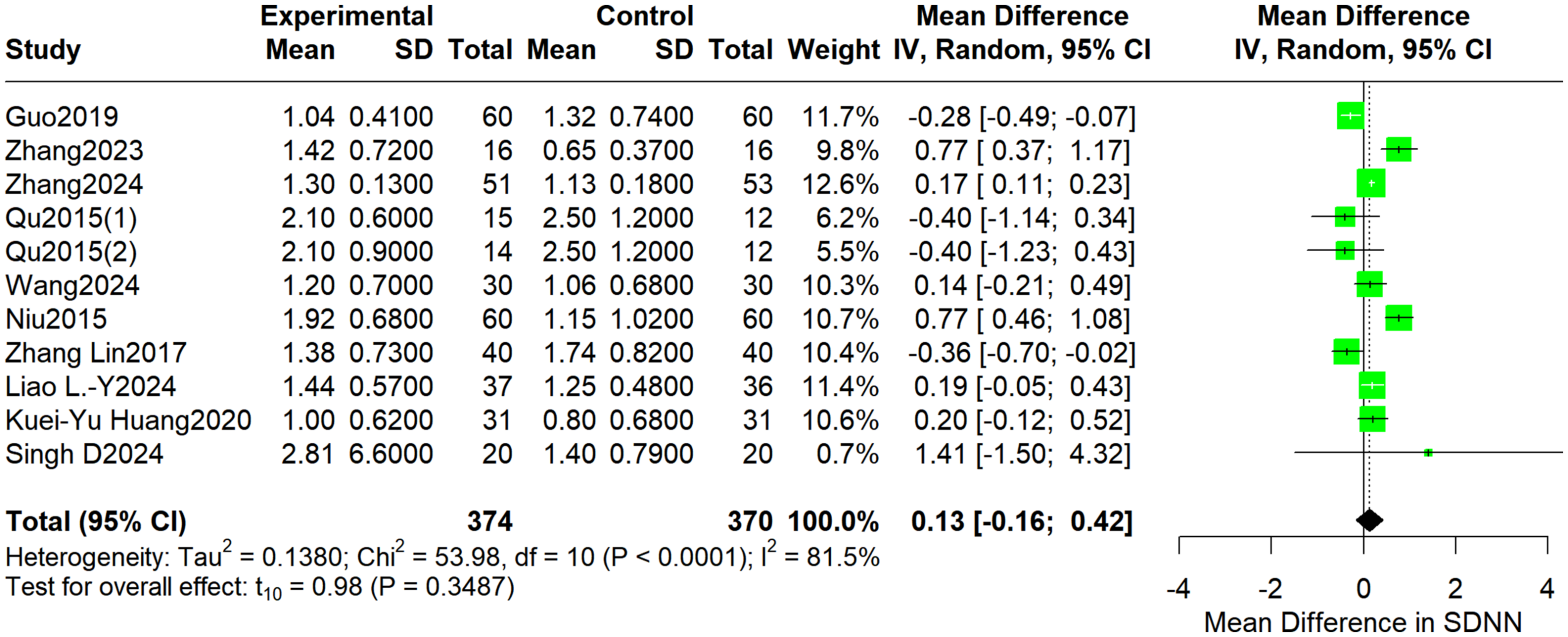
Forest plot depicting differences in the LF/HF ratio between acupuncture treatment and control interventions.

For the LF/HF ratio, the pooled MD was 0.13 (95% CI: −0.16 to 0.42; I^2^ = 81.5%, *p* = 0.3487) (Figure 6).

### Treatment-related adverse events

**Figure 7:**
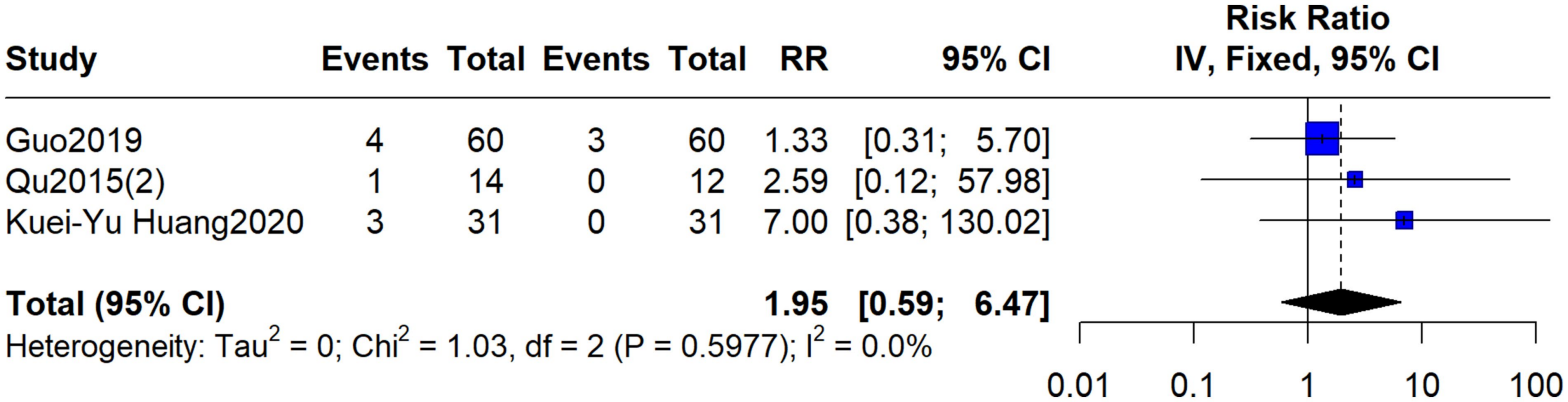
Forest plot comparing adverse events between acupuncture and control treatments for autonomic nervous function.

Three studies reported treatment-related adverse events. Eight events were reported in the acupuncture group: local hematoma (*n* = 2), numbness (*n* = 1), skin allergy (*n* = 1), dizziness (*n* = 1), and needle fainting (*n* = 3). All adverse events were mild, resolved during treatment with appropriate management, and no serious adverse events were reported in any study. Three adverse events were reported in the control group. The pooled RR was 1.95 (95% CI: 0.59–6.47; I^2^ = 0%, *p* = 0.5977) (Figure 7).

### Sensitivity analyses

Sensitivity analyses were performed for all efficacy outcomes (excluding adverse events), as detailed in Table 2. The analysis indicated that the heterogeneity for HF was primarily influenced by the study by Liao et al. (Liao et al., 2024); exclusion of these studies markedly reduced heterogeneity. The heterogeneity for LF, SDNN, and LF/HF remained relatively stable, with no single study markedly altering the results upon exclusion, suggesting dispersed or multifactorial sources of heterogeneity. The I^2^ statistics were predominantly above 30%, indicating moderate to high heterogeneity.

**Table 2:** Sensitivity analyses of HRV parameters.

Removing studies	I^2^ (SDNN)		I^2^ (LF)		I^2^ (HF)		I^2^ (LF/HF)	
	Before removing	After removing	Before removing	After removing	Before removing	After removing	Before removing	After removing
Guo 2019	96%	97%	95%	95%	41%	40%	84%	80%
Zhang 2023	96%	97%	95%	95%	41%	47%	84%	84%
Zhang 2024	96%	97%	95%	91%	41%	43%	84%	81%
Qu 2015(1)	96%	97%	95%	95%	41%	44%	84%	85%
Qu 2015(2)	96%	97%	95%	95%	41%	33%	84%	85%
Wang 2024	96%	97%	95%	95%	41%	47%	84%	86%
Niu 2015	96%	94%	95%	90%	41%	44%	84%	83%
Zhang Lin 2017	96%	95%	95%	95%	41%	41%	84%	83%
Liao L.-Y 2024	96%	96%	95%	95%	41%	7%	84%	86%
Kuei-Yu Huang 2020	96%	97%	95%	95%	41%	44%	84%	86%
Singh D 2024	96%	97%	95%	95%	41%	47%	84%	86%

### Publication bias

**Figure 8:**
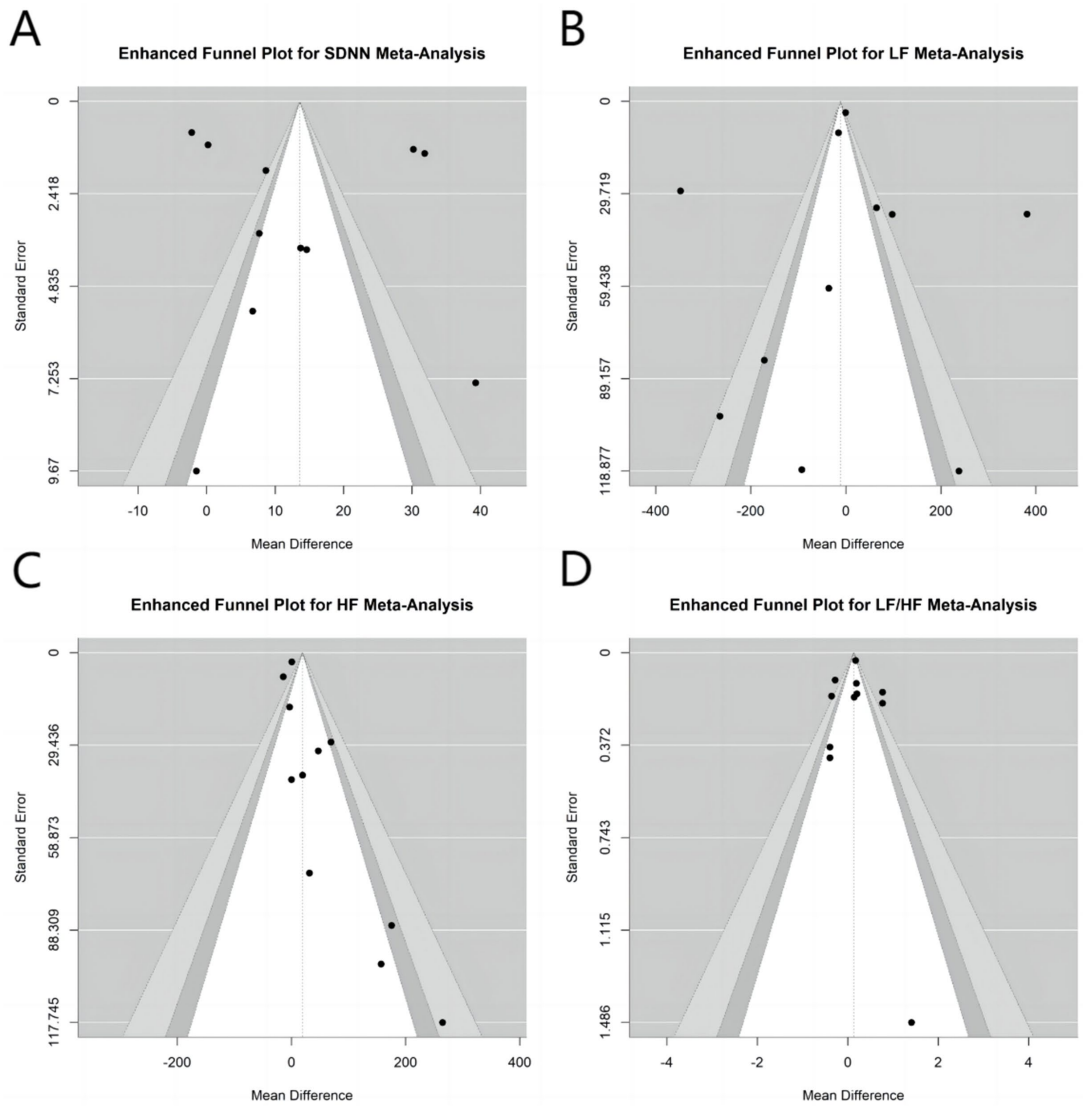
Funnel plots assess potential publication bias for acupuncture’s modulation of autonomic nervous function. **(A)** SDNN, **(B)** LF, **(C)** HF, and **(D)** LF/HF.

Funnel plots were utilized to assess potential publication bias for SDNN (Figure 8A), LF (Figure 8B), HF (Figure 8C), and LF/HF (Figure 8D).

## Discussion

Acupuncture, a therapeutic modality of CAM, involves the insertion of fine needles or the application of moxibustion at specific acupoints to stimulate and regulate physiological functions. It is used for managing diverse conditions, including pain, chronic diseases, and inflammation (Liu et al., 2025). With origins in China dating back over 3,000 years, acupuncture is widely utilized globally as a complementary therapy (Zhang et al., 2022). Its proposed mechanisms involve acupoint stimulation to restore bodily equilibrium, operating independently of pharmacological agents. The applications of acupuncture span digestive, immune, and neurological disorders, with its bidirectional regulatory capacity potentially beneficial in states of both immune suppression and overactivation (Liu et al., 2024). Although its mechanisms share conceptual parallels with the sympathetic-parasympathetic balance of the ANS, robust evidence specifically quantifying acupuncture’s efficacy in modulating ANS function has been limited. Our meta-analysis provides preliminary evidence suggesting that acupuncture may offer significant therapeutic advantages compared to other treatments, such as pharmacological interventions.

The core mechanisms underpinning acupuncture’s modulation of the ANS involve neural reflex pathways and neuro-immune interactions. First, somato-autonomic reflex pathways are pivotal; for instance, electroacupuncture at ST36 acupoint activates the vagus nerve, suppressing systemic inflammation, whereas stimulation at ST25 proves ineffective, indicating body region specificity (Liu et al., 2021). Second, acupuncture influences neurotransmitter release, such as stimulating hypothalamic melanin-concentrating hormone neurons to modulate ANS activity in Parkinson’s disease models (Liu et al., 2021). Furthermore, in instances of ANS dysregulation leading to insulin resistance, acupuncture can modulate neuronal excitability, thereby restoring hepatic glucose metabolic balance (Zsombok et al., 2024). Collectively, the mechanisms encompass reflex pathway activation, neuroendocrine regulation, and bidirectional control of the immune system, forming a multi-tiered regulatory network.

The findings from these clinical studies provide clues to the potential mechanisms by which acupuncture modulates the autonomic nervous system (ANS), likely involving neural reflexes and systemic regulation. At the level of neural reflex, studies on generalized anxiety disorder (GAD) and mild hypertension both observed that acupuncture intervention was accompanied by a significant increase in the high-frequency (HF) power of heart rate variability (HRV) and the high coherence ratio of heart rhythm coherence (HRC). This phenomenon suggests that the effects of acupuncture may be associated with the activation of parasympathetic nervous pathways, primarily involving the vagus nerve (Meira do Valle and Hong, 2024; Liu et al., 2015). Regarding systemic and long-term regulation, the migraine prophylaxis trial demonstrated that true acupuncture was superior to sham acupuncture in reducing attack frequency, with effects sustained after treatment cessation. This finding implies that the effect of acupuncture is not merely a placebo and may involve sustained modulation of the central nervous system and autonomic integration functions (Zhao et al., 2017). Furthermore, in the hypertension study, the sustained reduction in blood pressure occurred alongside enhanced indicators of vagal activity, further supporting the hypothesis that acupuncture can influence cardiovascular function by modulating autonomic balance (Liu et al., 2015). Collectively, these findings point to the modulation of autonomic balance as a potential mechanism through which acupuncture exerts its therapeutic effects across different disorders.

Clinical research has corroborated acupuncture’s regulatory effects on the ANS across various patient populations. In patients with postprandial distress syndrome, acupuncture treatment, evaluated via RCTs, significantly improved symptoms, serum hormone levels, and ANS indicators such as heart rate variability, affirming its direct modulatory role on autonomic function (Kupari and Ernfors, 2020). In heart failure patients, where ANS dysregulation (e.g., sympathetic hyperactivity and parasympathetic withdrawal) is a central mechanism, acupuncture or nerve stimulation may improve outcomes, although evidence requires strengthening (Fu et al., 2024). Moreover, in long COVID-19 patients, ANS imbalance contributing to cardiovascular symptoms has drawn attention to modulation strategies such as slow-paced breathing, suggesting potential utility for acupuncture as an intervention (Tseng et al., 2025). Overall, clinical evidence supports the use of acupuncture in digestive disorders, heart failure, and viral infection sequelae, yet more precisely designed trials are warranted.

In this study, we selected SDNN, LF, HF, and LF/HF as evaluation metrics. Conventionally, both PNS and SNS activities are believed to contribute to LF power; PNS activity primarily influences HF power, and the LF/HF ratio measures sympathovagal balance. However, some researchers have questioned the validity of LF and LF/HF for assessing autonomic function (Mauro et al., 2025; Shaffer and Venner, 2013). Consequently, we additionally examined SDNN, a metric generally considered more reliable for evaluating overall autonomic modulation and sympathovagal balance (Billman, 2013; Martinez et al., 2024).

This study is notably challenged by significant heterogeneity, particularly observed in the highly inconsistent results for LF, HF, and LF/HF. Potential sources of heterogeneity include the following: first, the diverse range of diseases studied, which manifest ANS dysregulation through different forms and mechanisms; second, considerable variation in acupuncture intervention modalities (e.g., electroacupuncture vs. manual acupuncture, stimulation parameters); and third, inconsistency in HRV recording equipment, as not all studies utilized 24-h Holter monitoring. Nonetheless, sensitivity analysis identified specific sources of heterogeneity (e.g., the influence of the study by Liao et al., 2024, on HF), suggesting that future research should prioritize standardized measurement protocols and intervention regimens.

Contrasting with the null findings of this study (e.g., non-significant difference in LF/HF), some previous studies have reported positive regulatory effects of acupuncture on the ANS. Reasons for these discrepancies may include the lack of rigorous blinding in most included RCTs, which may have introduced bias; small sample sizes in some studies, limiting statistical power; and the susceptiblity of ANS measurements to interference from factors such as emotional state, environment (da Silva, 2015), and caffeine intake (Nunan, 2010), all of which may impact the stability of the results.

Although this study suggests potential benefits of acupuncture in modulating the ANS, the overall quality of evidence is low (mostly rated low or very low certainty according to GRADE). Safety data indicated a slightly higher incidence of adverse events in the acupuncture group compared to the control group, although this difference was not statistically significant. Furthermore, the interpretation of our findings must acknowledge the inherent methodological challenges in acupuncture clinical trials. First, the standardization of acupuncture interventions remains difficult; variations in acupoint selection and stimulation intensity across studies lead to difficulties unifying treatment protocols, which is likely a significant source of clinical heterogeneity. Second, due to the distinctive nature of acupuncture procedures, implementing perfect blinding, particularly for practitioners, is highly challenging and may introduce performance bias. Third, as an invasive procedure, acupuncture might elicit stress responses in some participants due to needle-related anxiety or fear, a psychophysiological effect that could potentially confound the immediate measurement of autonomic nervous function outcomes, such as heart rate variability. Finally, the ongoing academic debate regarding the interpretation of frequency-domain HRV indices (e.g., LF and LF/HF) for assessing autonomic function adds another layer of uncertainty to the interpretation of our results. Therefore, future rigorously designed, large-sample, multicenter RCTs are essential to further validate the modulatory effects of acupuncture on the ANS and its clinical implications. Future research should focus on standardizing acupuncture protocols, harmonizing ANS assessment tools, and implementing long-term follow-up to evaluate the sustained effects of acupuncture on ANS function. Moreover, integrating multidimensional indicators from neuroimaging and molecular biology will help elucidate the mechanisms of acupuncture in ANS modulation, thereby advancing its clinical application in autonomic nerve-related disorders.

## Conclusion

This meta-analysis provides preliminary evidence that acupuncture may modestly improve autonomic nervous function, particularly global HRV as reflected by SDNN, with a favorable safety profile. These findings support the consideration of acupuncture as a complementary therapy for conditions involving ANS imbalance. However, given the methodological limitations and heterogeneity of existing studies, rigorously designed trials are warranted in the future to confirm these effects and establish standardized treatment protocols for clinical implementation.

## Data Availability

The original contributions presented in the study are included in the article/supplementary material, further inquiries can be directed to the corresponding authors.
